# Gene Therapy for Advanced Melanoma: Selective Targeting and Therapeutic Nucleic Acids

**DOI:** 10.1155/2013/897348

**Published:** 2013-03-25

**Authors:** Joana R. Viola, Diana F. Rafael, Ernst Wagner, Robert Besch, Manfred Ogris

**Affiliations:** ^1^Pharmaceutical Biotechnology, Department of Pharmacy, Ludwig-Maximilians-Universität, Butenandstraße 5-13, Munich, Germany; ^2^Department of Nanomedicine and Drug Delivery Systems, Faculty of Pharmacy, iMED.UL, Research Institute for Medicine and Pharmaceutical Sciences, University of Lisbon, Avenida Professor Gama Pinto, Lisbon, Portugal; ^3^Center for NanoScience (CeNS), Ludwig-Maximilians-Universität, Munich, Germany; ^4^Department of Dermatology and Allergology, Ludwig-Maximilians-Universität, Munich, Germany

## Abstract

Despite recent advances, the treatment of malignant melanoma still results in the relapse of the disease, and second line treatment mostly fails due to the occurrence of resistance. A wide range of mutations are known to prevent effective treatment with chemotherapeutic drugs. Hence, approaches with biopharmaceuticals including proteins, like antibodies or cytokines, are applied. As an alternative, regimens with therapeutically active nucleic acids offer the possibility for highly selective cancer treatment whilst avoiding unwanted and toxic side effects. This paper gives a brief introduction into the mechanism of this devastating disease, discusses the shortcoming of current therapy approaches, and pinpoints anchor points which could be harnessed for therapeutic intervention with nucleic acids. We bring the delivery of nucleic acid nanopharmaceutics into perspective as a novel antimelanoma therapeutic approach and discuss the possibilities for melanoma specific targeting. The latest reports on preclinical and already clinical application of nucleic acids in melanoma are discussed.

## 1. Introduction

Melanoma derivates from melanocytes—pigment cells of the skin. Melanoma most commonly arises from epidermal skin melanocytes (cutaneous melanoma), but primary tumors can also be found lining the choroidal layer of the eye (uveal melanoma) or the mucosal surfaces of the respiratory, genitourinary, and gastrointestinal surfaces. Similar to other tumors, the progression stage of melanoma is predictive for therapeutic success. Early stage melanomas (thin tumors) result in a 97% 5-year survival rate of the patients, after surgical removal [1]. Conversely, advanced melanoma patients, comprising metastasis in regional lymph nodes or other organs, face 5-year survival rates of less than 10% [1]. Due to the intrinsic tendency of melanoma to early metastasis, even small primary tumors have already led to metastasis and a substantial portion of diagnosed melanoma cases are of late progression stages. Treatment of advanced or metastatic melanoma has proven a challenge, as the conventional therapeutic approaches failed to translate into improved or significant survival rate in phase III clinical trials. Newer treatments were established in the last years that elicit unprecedented response rates in late stage melanoma, for example, up to 80% in the case of BRAF inhibitors. However, almost all tumors become resistant within months, and the treatment is available only for a subset of melanomas. Altogether, despite substantial improvements in therapeutic options during the last years, there is still an urgent need for alternative approaches.

Based on clinical and histopathological features melanoma cancer cells undergo four sequential phases before reaching metastasis [2]. These phases ensue from several genetic, epigenetic, and microenvironmental, modifications [3]. In the last decade, a number of reports have brought significant insight into melanoma genetics and molecular markers, which are essential for the development of therapies, and in particular targeted regimens. This paper will focus on melanoma targeted gene delivery; we aim at providing a general view on melanoma-targeting ligands, and other forms of specifically driving gene expression, reported in the literature, as well as review the most recent and/or relevant nucleic acid therapeutics employed in this field. The current paper will not dwell upon melanoma mutations or cancer transcriptional regulators (for reviews, see [4, 5]). Instead, the following melanoma section serves rather as a comprehensive overview on the key players of the neoplasia, which is essential for the understanding of targeted therapies.

## 2. From Melanocytes to Metastatic Melanoma

### 2.1. Four Steps Separate Melanocytes from Metastatic Melanoma

Presently, it is generally believed that melanomagenesis instigates from alterations in multiple molecules or pathways rather than a single high-risk melanoma loci. Moreover, melanoma progression is a dynamic process involving several steps, each requiring the activation of different genes. First, normal melanocytes undergo genetic alterations that lead to their transformation into benign nevi. Benign nevi differ from normal melanocytes in that they have initially proliferated in the basal layer of the epidermis; however, they entered a long-term dormant status due to the lack of additional oncogenic alterations. For example, the most frequent activating mutation in the BRAF gene occurs in the same frequency in nevi, where it causes a dormant status called oncogene-induced senescence [6]. Additional alterations then allow bypassing senescence leading to continued tumor cell proliferation. This progression stage is characterized by noninvasive horizontal growth and spread through the epidermis and has been termed as radial growth phase (RGP). Further transformation is required for invasive tumor growth from the epidermis into the dermis. This phase has been termed as vertical growth phase (VGP). For invasion, alterations like loss of adhesive molecules together with an increase in extracellular matrix degrading enzymes are characteristic. For metastasis, cell populations have to migrate to distant locations. For this, cells have to acquire more alterations that enable the complex processes underlying metastasis. These processes involve tissue invasion, entering, and evasion of blood or lymphatic vessels to reach distant location but also survival and proliferation at distinct locations. Hence, melanocytic cells have to become largely independent from their normal microenvironment [7].

### 2.2. Melanoma Progression: Risk Factors and Biological Drivers

**Figure 1:**
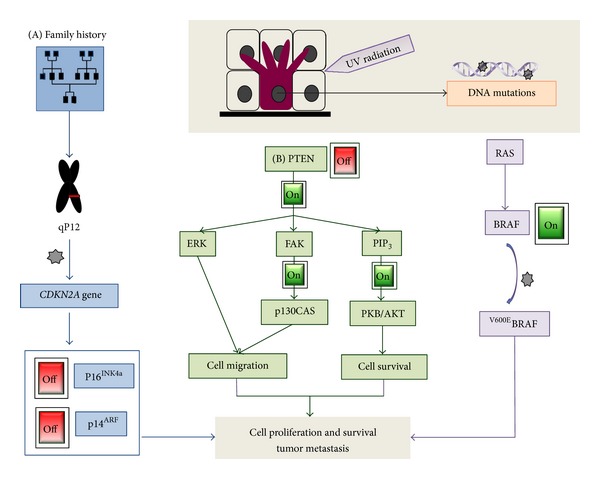
Schematic summary of the most common mutations found in melanoma patients. The most common risk for melanoma is UV, and most DNA alterations are typically UV-induced. Family history of melanoma accounts for a two-fold risk increase, through mutations at the level of CDKN2A gene. These often affect the tumor suppressors p16INK4a or p14ARF, which have roles in the cell cycle and apoptosis, respectively. On the other hand, there is the RAS/RAF signaling pathway, which importance is underlined by the fact that exclusively NRAS or BRAF is mutated in melanoma. However, the presence of BRAF mutations in benign nevi suggest that BRAF *per se* does not suffice for the tumor progression. Often mutations in PTEN pathways have been found to cooperate with RAS/RAF to reduce RAS/RAF-mediated senescence.

The most important risk factor for melanoma is UV irradiation upon sun exposure. Whole genome sequencing revealed that melanoma is the tumor type with the most DNA mutations—many being typical for UV-induced mutations [8]. Despite the plethora of DNA alterations, two gene mutations were found to be rather common in melanoma. A general overview on these mutations and their key players are schematically represented in Figure 1.

With respect to mutation frequency, the mitogen-activated protein kinase (MAPK) pathway plays a central role in melanoma. Activation of growth factor receptors leads to activation of RAS molecules which activate in a downstream phosphorylation cascade RAF, MEK, and ERK kinases. ERK kinase phosphorylates a panel of substrates leading to increased cell proliferation and survival. RAS molecules, comprising HRAS, KRAS, and NRAS, are small GTPases or G proteins, and activating mutations in NRAS are found in 10%–20% of melanomas. RAS molecules activate RAF family members consisting of ARAF, BRAF, and CRAF. A single nucleotide mutation in BRAF at amino acid 600—whereupon a valine (V) aminoacid is replaced by glutamic acid (E)—represents the most common mutation in BRAF. This mutant ^V600E^BRAF leads to an alternative protein structure and to a constitutive active protein. 50%–60% of melanomas contain an activating mutation in BRAF [9]. The outstanding importance of the RAS/RAF signaling pathway is documented by the observation that BRAF and NRAS mutations—exclusively NRAS or BRAF is mutated in a tumor—together are found in over 80% of melanomas and by inhibitors of mutated BRAF that are clearly effective in melanoma therapy.

Interestingly, ^V600E^BRAF has also been reported in melanocytic nevi [10–12], which rarely develop into melanoma. Nevi are described to be senescent, and, similarly, expression of ^V600E^BRAF in melanocytes induces oncogene-induced senescence [6]. These findings imply that BRAF mutations are involved in the first transition state of melanoma progression. Hence, this mutation *per se* is insufficient to drive tumorigenesis, rather additional alterations are required to avoid dormancy. 

Several pathways have been shown to cooperate with RAS/RAF signaling and to reduce RAS/RAF-mediated senescence. DNA damage due to oncogene-induced DNA replication stress has been proposed as an important mechanism of senescence [13]. Accordingly, molecules involved in DNA damage signaling have been shown to promote oncogenesis together with BRAF, for example, the loss of p53 [14]. Most evidence for BRAF cooperation exists for phosphatase and tensin homolog (PTEN). *PTEN* is a tumor suppressor gene that negatively modulates signal transduction via phosphatidylinositol phosphatase (PIP_3_, a cytosolic second messenger). This gene encodes for a lipid protein phosphatase that regulates cell growth and survival. Allelic loss or altered expression of PTEN can be observed in tumors. In melanoma, this lost/modified expression is present in 20%/40% of melanoma tumors, respectively [15, 16]. In a mouse model, it was shown that expression of ^V600E^BRAF in melanocytes leads to benign lesions that do not progress to melanoma. However, when PTEN was silenced, these mice developed metastatic tumors with high penetrance [17].

Regarding the family history of melanoma, a two-fold risk increase has been reported [18], and it was associated to the 9p12 chromosome [19]. In 1994, the cyclin-dependent kinase N2A (*CDKN2A*) gene was identified [20], and it is now hold as a high-risk melanoma locus. The *CDKN2A* gene encodes for two tumor suppressor proteins, p16^INK4a^ and p14^ARF^, involved in cell cycle and apoptosis, respectively. Explicitly, p14^ARF^ directly promotes the degradation of human double minute 2 (MDM2). MDM2 promotes ubiquitinylation and proteasomal degradation of p53. Accordingly, inactivation of p14^ARF^ leads to increased MDM2 levels leading to increased degradation of p53 [21]. The other product of the CDKN2A locus, p16^INK4a^, prevents cell cycle progression by binding to CDK4/6 and through a series of events prevents the release of E2F1 (a transcriptional inducer of S-phase genes) [22]. Mutations of p16^IK4a^, and similarly of *CDK4* gene [23, 24], can therefore lead to increased cell cycle progression. However, despite the contribution of *CDKN2A* mutations for oncogenesis, the absolute risk of melanoma in mutation carriers is still highly shaped by environmental and pedigree factors [25]. In close relation to pedigree structure is skin pigmentation; the positive connection between light skin color and melanoma risks is well known. Melanocortin-1 receptor (MC1-R) is responsible for the cutaneous pigmentation, and, interestingly, it has been reported as being overexpressed in both melanotic and amelanotic melanomas [26]. There are two forms of epidermal melanin: eumelanin (with a black-brown color) and pheomelanin (red-yellow color). The synthesis of eumelanin—in charge of UV attenuation—is stimulated by the activation of the MC1-R, through the binding of the tridecapeptide *α*-MSH, or *α*-melanocortin stimulating hormone [27–29]. The binding of *α*-MSH results in an increment of cAMP, which in turn upregulates the microphthalmia-associated transcription factor (MITF) inducing the transcription of pigment synthetic genes and the production of eumelanin. In addition, some MC1-R variants have been associated to melanoma risk [30]. *MITF*, on the other hand, is also involved in the regulation of the cell cycle and proliferation, and few variants of the gene have been found in melanoma patients [31, 32]. In particular, MITF(E318 K) was reported to represent a gain-of-function allele for the gene, supporting *MITFs* role as an oncogene. However, MITFs expression in melanoma metastasis is yet to be clarified, as there are also studies showing that downregulation and ablation of this gene create a more invasive phenotype *in vitro* [33] and increase tumor growth *in vivo *[34], respectively.

The transcription factor activator protein-2*α* (AP2*α*) has been suggested as a major key player in the transition from RGP to VGP [4]. Similar to several other mediators, AP2*α* also modulates a variety of cellular processes, including cell growth and apoptosis. In tumors, AP2*α* acts as a tumor suppressor, and high cytoplasmatic to nuclear expression ratio was shown to correlate with poor patients' prognosis [35, 36]. In particular, the promoters for the adhesion molecule MCAM/MUC18 [37], which is overexpressed in tumors, and tyrosinase kinase receptor, c-KIT (silenced in 70% of metastatic tumors) [38], have AP2*α* binding sites. AP2*α* has been described to directly bind to MCAM/MUC18 promoter and to inhibit its transcription, whereas it promotes c-KIT expression. Therefore, the loss of this transcription factor during melanoma results in high MCAM/MUC18 levels and c-KIT downregulation. In addition, the loss of AP2*α* was also appointed as a probable cause for the upregulation of the G-protein-coupled receptor protease activated receptor-1, PAR-1 [10, 39]. In PAR-1 promoter region, there are two binding complexes for AP2*α* and SP1. In normal melanocytes, AP2*α* binds to PAR-1 inhibiting its transcription. However, upon melanoma progression, the levels of AP2*α* decrease, and SP1 binds to the PAR-1 promoter instead, driving its expression. RAS, phosphoinositide-3 kinase (PI3 K), and MAPK pathways are all signaling events downstream PAR-1, and hence closely related to tumor progression [40].

During the metastatic process, following evasion into the blood circulation, tumor cells adhere to the endothelium at distant sites, and herein adhesion molecules are necessary. Together with selectins, integrins have been found to play crucial roles in these steps. Integrins are a family of transmembrane glycoproteins that mediate cell-cell and cell-matrix adhesion. It is therefore expected that their expression pattern changes during tumor growth, metastasis, and angiogenesis. In particular, *α*_v_ *β*_3_ and *α*_4_ *β*_1_ (very late activation antigen-4, VLA-4) have been reported as overexpressed in numerous cancer types [41, 42] and have served as therapeutic targets. VLA-4 has been shown to be used by malignant melanoma cells to adhere to the endothelium (binding to the ligand VCAM-1) [43, 44], and to promote transmigration [42, 45] and metastasis [46, 47].

## 3. Shortcomings of Current Melanoma Therapies

Overall, melanoma incidence has been increasing over the years, reaching an annually increase of 3.1% during the past two decades [48]. Early prognosis permits 90% survival rates by surgical removal. Yet, unresectable advanced melanoma is characterized by an aggressive behaviour, fast spread and metastasis, and a strong resistance to chemotherapy. Therefore, and in spite of the extensive research, the current prognosis for patients with advanced melanoma is limited. The earlier conventional chemotherapeutic treatment approved by US Food and Drug Administration (FDA), Dacarbazine, results in less than 10% response rate with median response durations of 4–8 months [49]. Alternative chemotherapeutic agents include Fotemustine, Temozolomide, Paclitaxel (often in combination with carboplatin), and Docetaxel [50]—all not yielding larger progression-free survival (PFS) or overall survival (OS) than Dacarbazine [50, 51]. Generally, chemotherapeutics suffer from a lack of targeting specificity; their low molecular mass results in easy and fast body secretion, and thus the need of increased doses, which leads to inevitable toxicity. Similarly, immunotherapy based on interleukine 2 (IL-2)—also FDA approved—has comparable response rates, and it is further restricted by the ensuing multiorgan toxicity, requiring management in specialized cancer centers. Although combined therapies resulted in higher response rates, they still failed to translate into improved survival, with no impact on PFS or OS compared to Dacarbazine alone [1, 52]. Another alternative is the combined treatment with the cytokine TNF*α* in combination with the alkylating drug melphalan. Although highly successful, this treatment is limited to local treatment of melanoma in-transit metastases in limbs by isolated limb perfusion due to live threatening systemic toxicity of therapeutically active TNF*α* doses [53].

In the last decade, much progress was achieved due to the discovery of mutations in the BRAF gene. This led to the development of therapies interfering with RAS/RAF signaling and to specific BRAF inhibitors. In August 2011, an alternative melanoma regimen, for patients positive for BRAF mutations, was brought into the market with the FDA approval of Vemurafenib (Zelboraf, Plexxikon/Roche). In Phase II and III studies, Vemurafenib showed a response rate up to 50%, yet the response duration varied between the phase studies [54–56]. In addition, Vemurafenib induces acanthopapillomas, keratoacanthomas, and cutaneous squamous cell carcinomas in the early treatment [57, 58]. Unfortunately, these unprecedented response rates are limited by the fact that almost all tumors become resistant to this therapy and the overall survival of patients was 6.7 months [59]. In addition, the treatment is only available for 50%–60% of patients with mutated tumors because it is not effective in tumors with wildtype BRAF. Nevertheless, this success has led to the development of other RAS/RAF pathway inhibitors, for example, for mutated BRAF or downstream kinases like MEK. Alternative activation of RAS/RAF pathway has been proposed as a resistance mechanism [60]. In line with this, the combination of BRAF inhibition with MEK inhibition led to an improved survival of 9.4 months [61].

Other new therapies that add to the therapeutic options for melanoma patients are immunotherapies. An anti-CTLA-4 antibody (Ipilimumab) improved survival of stage II and IV melanoma patients (10.1 versus 6.4 months) [62]. Cytotoxic T-lymphocyte Antigen 4 (CTLA-4) inhibits T-cell responses and respectively, CTLA-4 blockade promotes immune responses and antitumor activity. In an early analysis of anti-PD-L1 antibody, a 20% response rate in melanoma was observed. Importantly, these responses lasted for more than 1 year [63]. Similar to CTLA4, PD-1 reduces immune activation, and its inhibition can lead to reactivation of immune responses.

Altogether, even with respect to the recent advances in melanoma therapy, the high resistance rates and the restriction to certain patient subgroups demonstrate that there is still an urgent need to develop alternative therapies.

## 4. Assets of Nucleic Acid Nanoparticles in Antitumoral Approaches

As also observed for other tumor entities, melanoma treatment with low molecular weight chemotherapeutic drugs often results in the rise of resistant cancers cells, especially in case of relapsed disease. A well-known mechanism of resistance is the elevated expression of multidrug transporter proteins, like p-glycoprotein, which actively pump chemotherapeutics out of the cell [64]. Here, macromolecular approaches can be a suitable approach to overcome such resistance. As an example, the attachment of chemotherapeutics to polymers via reversible covalent bonds helps to overcome this type of resistance (for a recent review see [65]). Also, biotherapeutics, such as antibodies, have been successfully applied in melanoma therapy (see above), but also here resistance can occur, for example, when blocking of one cellular pathway responsible for cancer cell proliferation can be replaced by another [66]. In this case, the application of therapeutically active nucleic acids comes into play. Firstly, they exhibit a relatively high molecular weight, which prevents resistance mediated by p-glycoprotein upregulation. Secondly, nucleic acids can be designed to affect only malignant cells, for example, by using promoter elements being only activated in tumors, or as RNA oligonucleotides (like siRNA), which will enable the knockdown of a specific protein overexpressed in tumor tissue. Furthermore, the delivery of more than one siRNA targeting different pathways can prevent tumor resistance by blocking different resistance or escape strands. Last but not least, nucleic acid delivery permits systemic delivery of toxic agents, such as diphtheria toxin A [67] or tumor necrosis factor (TNF) [68], as they only become toxic after transcription in the target cell.

Solid tumors exceeding a certain size rely on a functional blood supply for access to nutrients and oxygen. In contrast to nonmalignant tissues, tumor vasculature often exhibits a leaky appearance, which in principle also allows nanosized particles to reach tumor cells [69]. Being packed into nanoparticles or polyplexes, nucleic acids can be protected from nucleases which are present in the bloodstream. Nevertheless, systemic delivery of nanopharmaceutics offers several pitfalls and obstacles, such as aggregation with blood cells, undesired adherence to the vessel wall, or opsonization with plasma proteins followed by clearance through tissue macrophages (a key component of the reticulo-endothelial system). Blood proteins interact both with negatively and positively charged nanosystems, whereas a neutral surface charge enables, in principle, blood circulation, as it has been shown for small nanocrystals, so called quantum dots [70]. Alternatively, nanosystems can be decorated with hydrophilic polymers, which, owing to their excessive hydration, shield the particles' surface charge, hereby preventing the aggregation with protein components. From the group of hydrophilic polymers, like N-(2-hydroxypropyl)methacrylamide (HPMA) [71], hydroxyethyl starch (HES) [72], or polyethyleneglycol (PEG) [73], PEG is the most commonly used one. In addition, targeting entities can be used to direct the nanocarrier to specific cells. Commonly, these are ligands that bind to receptors, or other cell surface molecules, that are overexpressed in tumor cells.

**Figure 2:**
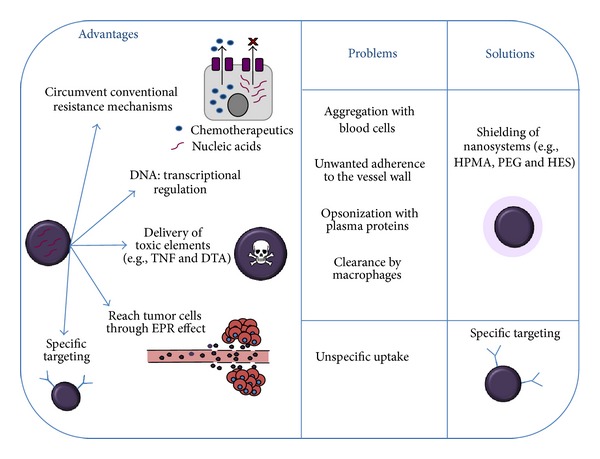
Advantages and limitations in nucleic acid nanosystems delivery. Particular advantages of nucleic acid therapies are (1) the ability to include tissue specific targeting (or transcriptional targeting) and (2) the possibility to systemically deliver genes encoding for proteins with toxic properties. Moreover, as macromolecules, nucleic acids can overcome resistance mechanisms such as that supported by p-glycoprotein. However, nucleic acids are vulnerable in blood circulation, and hence they must be protected against enzyme degradation and condensed in the form of polyplexes. Physiological barriers, such as reticulo-endothelial system, still present a threat for nanosystems, and these must be armed against possible interactions with blood cells that can result in opsonization or undesired blood vessel adhesion. Decoration of nanocarriers with PEG or HPMA can provide shielding effect, while decoration with ligands that can bind receptors overexpressed in tumors can assist in cellular targeting and internalization. TNF: tumor necrosis factor; DTA: Diphteria toxin A; HPMA: N-(2-hydroxypropyl)methacrylamide; PEG: polyethylene glycol; HES: hydroxyethyl starch.

Macromolecular drugs, which exceed the renal excretion limit and are able to circulate in the blood stream, can benefit from the so-called enhanced permeability and retention (EPR) effect: nanopharmaceutics accumulate in tumor tissue as they can penetrate the leaky vasculature but are retained within the tumor tissue due to incomplete lymphatic drainage [98]. This tumor deposition is a prerequisite for all steps that follow: binding to and internalization of the particles into target cells. The latter can be promoted by the incorporation of the earlier mentioned cell-binding ligands into the carrier system.  Figure 2 summarizes the limitations in nucleic acids delivery, the solutions for such limitations, and the therapeutic advantages of nucleic acid nanosystems.

## 5. On the Footsteps of Metastatic Melanoma: Cell Surface and Transcriptional Targeting

Directed approaches are of special interest as they have the potential to specifically distress malignant cells causing increased local concentrations of the active agent and avoiding undesired side effects. Tracking down melanoma-associated molecular targets involves identifying signaling pathways' key players, earlier described, as much as cancer cell surface markers. In particular, for gene therapy, cell surface markers are important, and these abide with the conception of a treatment addressing multiple melanoma subgroups—as cells with different mutations can still exhibit common surface markers. Ergo, it is crucial to identify critical and idiosyncratic targets for these cells.  Table 1 summarizes the most common melanoma-targeting tools herein described.

Already reported in the early seventies [99], one of the largely explored targets is the melanocortin-1 receptor (MC1-R), which is also overexpressed in numerous melanoma cases. MC1-R belongs to a class of G-coupled protein receptors (MC1-R–MC5-R), where the different receptors allocate in different tissues, reflecting their functions. While MC1-R is found in hair and skin [100], MC2-R is localized in adrenal glands [101], whereas MC3-R and MC4-R are in hypothalamus [102] and MC5-R in kidneys [103]. However, owing to their similarity their binding domains may share common affinities, and certain peptide motifs can bind to several receptors [74]. For targeting purposes, the most well-known and used MCR-1 ligand is the synthetic [Nle^4^, D-Phe^7^]-*α*-MSH or NDP-*α*-MSH [75]. The substitution of methionine in position four by norleucine (Nle^4^) and of phenylalanine for its d-counterpart in position seven (d-Phe^7^) renders this peptide with higher affinity and resistance to enzyme degradation than its native form. However, NDP-*α*-MSH was shown to have a strong nanomolar binding affinity towards MC3-R, MC4-R, and MC5-R [74], and, for gene delivery, it is crucial to decrease off-target effects. Aiming at the design of ligands suitable for micelle conjugation, and with an adequate selectivity to MC1-R, Barkey et al. have conducted a comparative study in which they screened several candidate ligands [74]. This paper allowed the following conclusions: (1) free rotation of carbons that compose the peptide's biding motif seems to be required for MC1-R avidity; (2) alkyl modifications, for the attachment of triblock polymer micelle, at the N-terminal of the peptide, did not affect binding affinity in the short four amino acid peptide; (3) for peptides twice as long, C-terminal modifications for micelles' attachment did not altered binding affinities. In addition, the authors have synthesized micelles conjugated to the short peptide version [4-phenylbutyril-Hist-dPhe-Arg-Trp-Gly-Lys(hex-5ynoyl)-NH_2_], through a PEG linker. And importantly, *in vitro* cell-uptake studies showed the ability of conjugated micelles to selectively bind to MC1-R receptor, and, whether due to multivalent interactions or other factors, the micelles had higher avidity for the receptor than the ligand alone. Nevertheless, further studies (i.e., by flow cytometry or confocal laser microscopy) to quantify the uptake of these conjugated micelles are needed to better evaluate the delivery efficiency of this platform. More recently, *α*-MSH peptide has been conjugated to a nanoplatform based on the heavy chain of the human protein ferritin (HFt) [76]. Ferritin can be used to build a hollow nanocage that can transport materials such as Fe_3_O_4_, Co_3_O_4_, Mn_3_O_4_, Pt, and Au and hence be used for imaging and therapeutic purposes. The targeted ferritin nanocages have been evaluated *in vitro *and *in vivo*. Unfortunately, the authors have not analyzed the *in vivo* distribution of their nanoparticles, and the targeting efficiency was evaluated by immunohistochemistry in the tumor tissue in relation to normal skin. In a similar approach to that of HFt nanocages, Lu and collaborators have used hollow gold nanospheres, conjugated to NDP-*α*-MSH, aiming at cancer photothermal ablation [77]. In this study, nude mice were subcutaneously inoculated with B16/F10 murine melanoma cells, and the nanoparticles were administered intravenously. The authors have collected different organs and were able to show the targeting effect by the NDP-*α*-MSH-gold nanospheres.

Interestingly, targeting of MC1-R by *α*-MSH peptide has been mostly used in radionuclide therapy studies and for diagnostic purposes. Currently, 2-[^18^F]fluoro-2-deoxy-d-glucose (^18^F-FDG) is the only radioactive probe used in the clinic to detect melanoma. Be that as it may, ^18^F-FDG is an unspecific positron emission tomography (PET) imaging agent with poor sensitivity towards micrometastatic sites [78, 79], a fact that underlines the general insufficiency in melanoma targeting.

Regarding MC1-R targeting, Yubin Miao and Thomas P. Quinn's extensive work is of particular interest, reporting on two generations of an NDP-*α*-MSH-based peptide used for melanoma imaging by single-photon emission-computed tomography (SPECT) and more recently by PET. What distinguishes the two *α*-MSH peptide generations is mostly the peptide's length, being twelve aminoacid-long in the first generation (CycMSH) [80–82] and six in the second (CycMSH_hex_) [83, 84]. In both generations, the peptide is cyclized (Cyc), and the MC1-R binding motif (His-dPhe-Arg-Trp) is conserved. The peptides have also undergone structural modifications concerning the aminoacid linkers, which are used to support the peptide cyclization and bridge the targeting ligand and the radiometal chelator. Interestingly, the authors have observed that the exchange of single aminoacids in these linkers [85], and the introduction of—GlyGly—linker between the chelator and the peptide [84] resulted in improved melanoma targeting, with decreased renal excretion and liver uptake of the radiolabelled peptide in B16/F1 melanoma-bearing C57 mice. These studies underscore the structural role of the targeting moiety but also of the integral component being delivered. In other words, the addition of a targeting entity to a carrier does not necessarily suffice for efficient deliver; the number of peptides conjugated to the delivery platform, the site of conjugation and the size and type of the linker play an important role.

Integrin targeting has also been extensively explored for cancer gene delivery in general. After the discovery of adhesion molecules as mediators of tumor metastasis, the identification of their binding motifs opened the possibilities for targeted therapies. Several peptide fragments have been employed to target these mediators, either as antagonists or as ligands for drug delivery purposes. One of the utmost targeted integrin is the *α*_v_ *β*_3_. *α*_v_ *β*_3_ plays a central role in angiogenesis—the formation of new vessels— and, by serving as receptor for extracellular matrix proteins, it mediates migration of endothelial cells into the basement membrane, and regulates their growth, survival, and differentiation. It is therefore no surprise that such integrin is found upregulated in different tumor cells, where it is involved in processes that govern metastasis. The integrin's binding peptide motif has been identified in 1990 [121]—Arginine-Glutamine-Aspartate or RGD—but studies that followed have shown that the cyclic version of RGD (cRGD) has higher binding affinities towards the integrin [86, 87]. Either alone or in combination with other ligands, cRGD has been conjugated to several nanocarriers for both diagnostic and therapeutic purposes [88–90].

Another integrin reported to have a dominant function in the metastatic spread is *α*_4_ *β*_1_ or VLA-4. Okumura, and more recently Schlesinger, have shown, in different settings, that inhibition of VLA-4 by natalizumab (an antibody against *α*_4_ integrin) significantly decreased melanoma lung metastases in murine models [42, 44, 122]. In 1991, Makarem and Humphries have identified the Leucine-Aspartate-Valine (LDV) sequence as the integrin's motif [123], and a few years later, Vanderslice et al. have reported on a series of cyclized peptides based on LDV that were assayed for the inhibition of the integrin [124]. However, and despite the numerous reports relating this agent to tumor metastasis, and to melanoma in particular, most of the literature relies on the LDV sequence as an antagonist, rather than for deliver purposes, where, to our knowledge, there is only one paper reporting on *in vitro* studies [91]. Indeed, VLA-4 is found in multiple leukocyte populations; VLA-4 is a vital receptor of leukocytes, and it is involved in the immune response. Hence, a systemic application of VLA-4 inhibitors, or binding peptides, could induce undesired partially immunosuppressive effects. In this context, the application of transcriptional-targeting strategies could potentially prevent off-target effects and prove this ligand a promising tool. In fact, tissue-specific elements as components of the DNA vector can provide a tight control over gene expression and complement and strengthen targeted-delivery. Commonly, tumor cells' surface markers entail receptors that are also present in nontumor cells but are rather overexpressed in their malignant form. This is the case for both the integrins here described, but also the transferrin receptor [92]—all used as melanoma targets. Therefore, off-target effects can occur, and for gene delivery purposes, tissue-specific control elements are an elegant way to bypass undesired side effects. These control elements consist of nucleic acid sequences that are recognized by proteins or other nucleic acids, which hereby regulate gene expression. For the case of melanoma, tissue specific promoters have been described, and these include tyrosinase [93–95] and melanoma inhibitory activity (MIA) [96, 97]. Gene expression is hence to be accomplished in tissues where such promoters are activated.

MicroRNA (miR) binding sites can also serve as transcriptional control elements. MicroRNAs are a class of short (20–22 nucleotides long) regulatory RNAs, which are believed to regulate as many as 30% of all genes. Several microRNAs are tissue-specific and fine-tune genetic circuits, some of which are critical for normal development, cellular differentiation, and normal cellular homeostasis. If the target sequence and microRNA have perfect complementarity, the mRNA is eliminated by a RNA degradation pathway. In the context of transcriptional control, this means that a DNA vector that contains specific miR-binding sites is only translated in cells where the miR in question is absent [125, 126]. In tumor cells, several microRNAs are deregulated, while miRs enrolled in cell homeostasis are downregulated those involved in cell proliferation and differentiation are upregulated [127]. For the case of melanoma, miR let-7b, miR-193b, miR-34a, miR-155, miR-205, miR148, miR-137, and miR-152 have been found downregulated (for a review on melanoma microRNAs, see [127]) and can therefore be suitable targets for transcriptional regulation when expressed in normal tissue.

## 6. Therapeutic Nucleic Acids in Melanoma

As opposed to conventional therapy, traditionally, that is, in the case of loss of function, gene therapy aims at permanent correction of a defected or missing gene by replacing with or providing, respectively, the corrected version—for example, by the introduction of plasmid DNA (pDNA). Ideally, this approach translates into a single treatment, or few initial treatments, rather than several (or life long) required to provide the patients with the functional form of the protein. However, this permanent correction treatment has proven very challenging.

In the last twenty years, new nucleic acids with attractive therapeutic properties were discovered, notably, siRNA and microRNAs. Small interference RNA (siRNA) has the ability to specifically silence protein expression—an asset particularly valuable for antiviral and cancer regimens. In general, also miRNA negatively regulates gene expression, although via two different mechanism depending on the degree of complementarity towards its mRNA target. Nucleic acid-based approaches offer several advantages when compared to treatment with small molecules or proteins. They can be seen as mostly inactive prodrugs, which are activated at the tumor site producing a therapeutically active protein or knocking down a specific target gene. Importantly, nucleic acid targeted delivery systems, preferably also relying in transcriptional targeting, decreasing off-target effects and toxicity, and permitting a systemic administration otherwise not feasible with a therapeutic agent with toxic properties.

**Figure 3:**
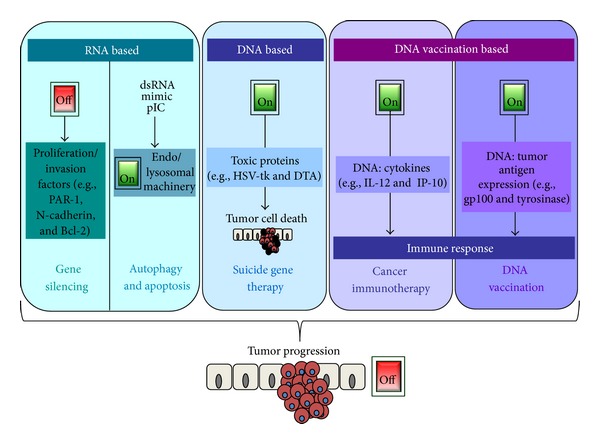
Different strategies used in antitumor nucleic acid approaches. RNA-based strategies are commonly used to downregulate agents that are upregulated to favor cell proliferation or migration, such as Bcl-2. Alternatively, double stranded RNA (dsRNA) mimic polyinosinic-polycytidylic acid (pIC) can be used to engage the endosomal machinery, resulting in autophagy and apoptosis. Conversely, pDNA delivery aims at the expression of a protein that can (1) have toxic properties, directly causing tumor cell apoptosis (pDNA-based approaches); (2) be a chemokine, thus recruiting cell-mediated immunity; or (3) be a tumor antigen, recruiting humoral immunity (DNA vaccination-based strategies). Ultimately, all strategies aim at putting an end to tumor progression and eventually tumor cell destruction.

In parallel with new therapeutic nucleic acid tools, the last two decades brought insight into tumorgenesis in general and unveiled a plethora of therapeutic concepts against cancer (Figure 3). The following paragraphs will deal with different antimelanoma approaches based on nucleic acids.

Despite the apparent tumor tolerance, humoral and cellular immune responses are naturally generated against tumor antigens. Hence, whether the tumor grows as a result of stealth and nonrecognition or as the result of escape and immunological shaping [128], its recognition by the immune system can still be prompted. Indeed, at a later stage, during the progressive growth phase, tumors may become more immune-activating for varies reasons: damage or disruption of surrounding tissue, generation of reactive oxygen species, upregulation of stress protective factors, or death by necrosis or apoptosis. However, at this stage, it is not known whether the tumor still needs to escape immune recognition, as it is unclear that these immune responses can cause tumor destruction [128]. Therefore, a number of studies have focused in eliciting earlier and suitable tumor recognition by the immune system. In a nucleic acid therapy context, this transliterates into genetic immunization or DNA vaccination: the delivery and transcription of a gene encoding antigens or immunestimulatory molecules that elicit an immune response. As an example, interleukine-12 (IL-12) has been used and studied in different animal models [104, 105]. IL-12 is originally produced by mononuclear phagocytes and dendritic cells and is responsible for activating NK and CD4^+^ T cells and inducing the production of high levels of interferon gamma (INF-*γ*). Interestingly, IL-12 has been described to increase antitumor immune responses [129, 130], and later studies investigated its suitability for a DNA vaccine approach against melanoma [106]. IL-12 effects appeared to be long lasting and efficient against tumor metastases, although not mainly mediated by INF-*γ* [106]. The murine studies also revealed moderate toxicity caused by IL-12, and while lower IL-12-encoding pDNA doses can be administered, ideally the gene expression should be controlled, regarding the tissue and the durability of the expression. Although DNA vaccination against a strong melanoma tumor antigen should be possible, the authors have not seen an effect on lung metastases when using melanoma-associated glycoprotein 100 (gp 100)/pmel17 pDNA alone. Adjuvants appear to be necessary for a successful DNA vaccination: the authors have seen an effect when the gp 100-pDNA was administered together with IL-12, similar to other murine study where granulocyte-macrophage colony-stimulating factor was used [107]. Alternatively, in a canine study, the developed vaccine was based on the human (rather than canine) gp 100 protein [108], where the human form of the antigen acted as adjuvant. Together with gp 100, and for the case of melanoma, two more tumor genes have been described for DNA vaccination: MART-1 and tyrosinase [108, 109].

Also, the expression of chemokines, such as monocyte chemoattractant protein-1 (MCP-1) and interferon-inducible protein-10 (IP-10), can mediate an immune response. In particular, IP-10 as been described by Sgadari et al. as an antitumor agent and found to promote damage in established tumor vasculature as well as tissue necrosis in a murine model for the human Burkitt lymphomas [131]. Based on this, and after their studies with IL-12, Keyser and collaborators have investigated the efficiency of IP-10-encoding pDNA therapy in murine melanoma models [110]. The authors have used two murine tumor models, whereupon cells have been injected subcutaneously (originating a solid tumor) or intravenously, inducing lung metastases. When administered alone, and intramuscularly (resulting in systemic circulation), IP-10-encoding pDNA showed an antimetastatic effect, reducing the number of lung metastases as compared to the control-pDNA treated group. When administered with IL-12-encoding pDNA, IP-10 pDNA enhanced the IL-12 effect, and decreased its earlier observed toxicity. This anti-neoplastic effect of IP-10 has been attributed to the engagement of NK cells and the inhibition of angiogenesis and cell proliferation.

Alternative antitumor strategies aim at a direct destruction of cancer cells, through the delivery of pDNA encoding for a toxic protein—DNA-based strategies. This is referred to as a suicide gene therapy or gene-directed enzyme prodrug therapy (GDEPT), when the nucleic acid sequence encodes for an enzyme, which is not directly toxic but instead converts a nontoxic prodrug into a cytotoxic metabolite. The first proof of principle of GDEPT was presented in the mid-eighties and involved the herpes simplex thymidine kinase (HSV-tk) and the prodrug ganciclovir (GCV) [132]. Presently, HSV-tk as well as other approaches, such as Diptheria toxin A chain (DTA), have been employed in the clinics, the most successful cases being reported in ovarian and prostate cancers [67, 133]. As for melanoma treatments, HSV-tk has been the most commonly used [111–113], although there is no human clinical trial yet. Suicide gene therapy has also been proven effective when used in combined approaches, such as with cytokine-enhanced vaccine in a clinical trial involving canine melanoma patients [134]. Despite promising, this strategy is currently restrained by a poor delivery; most nanocarriers are not as target-specific and efficient as required, and the toxic gene does not reach the tumor cells in efficacious concentrations.

A number of studies have instead focused on mediators of cell proliferation and differentiation, which are upregulated during tumorgenesis, aiming at their downregulation by means of siRNA delivery [114, 135–137]—these are RNA-based approaches. As an example, based on the fact that in epithelial cells, N-cadherin induces changes in morphology of a fibroblastic phenotype (rendering the cells more motile and invasive), the laboratory of Laidler has investigated the outcome of N-cadherin silencing in human melanoma cell lines [114]. Although the results suggest that N-cadherin positively affects the regulation of the cell cycle and proliferation through activation of the AKT kinase pathway, further investigations are needed to describe the mechanism. Similarly, Villares et al., upon the observation that thrombin receptor (or protease-activated receptor-1, PAR-1) is overexpressed in highly metastatic melanoma cell lines, has evaluated the therapeutic potential of siRNA against PAR-1 [115]. The authors have observed a significant reduction of *in vivo* tumor growth as well as in the number of metastatic lung colonies. This report showed that downregulation of PAR-1 decreased the expression of matrix metallopeptidase-2 (MMP-2), interleukin 8 (IL-8), and vascular endothelial growth factor (VEGF), resulting in an overall decrease in angiogenesis and blood vessels. In 2010, Davis et al. reported on the first human clinical trial (including three melanoma patients) on siRNA therapy against melanoma [92]. The siRNA targeted the M2 subunit of ribonucleotide reductase (RRM2), and the protein knock down was confirmed at the mRNA level but not corroborated to the same extend by the protein analysis. Nevertheless, the fact that the authors used a delivery vector targeting the transferrin receptor without showing analysis of such receptor expression in melanoma cells was left to be explained [138].

Of special interests are combinatorial strategies involving siRNA delivery as these, similar to other combinatorial therapies, cause the most significant outcomes. Particularly, Poeck and coauthors have used a simple and elegant siRNA design [116]. The authors targeted Bcl2 (an apoptosis regulator protein), which was reported to play a central role in the resistance of melanoma cells to chemotherapy [7, 116, 139, 140]. By adding 5′-triphosphate ends to their siRNA, the authors also activated innate immune cells, induced the expression of interferons, and caused specific cell tumor apoptosis. These actions are a consequence of the recognition of 5′-triphosphate ends by the cytosolic retinoic acid-induced protein-1 (Rig-1) and synergized with the silencing effects originated from siRNA resulting in massive tumor destruction in the murine lung metastases. Two years earlier, aiming at RNA-based vaccination, Tormo et al. first reported on a promising double stranded RNA (dsRNA) mimic polyinisine-polycytidylic acid (pIC) [117]. Importantly, the therapeutic effect of the dsRNA was significantly increased when delivered in the form of a complex, together with polyethyleneimine (PEI)-[pIC]^PEI^. Initially, the dsRNA mimic was thought to engage toll-like receptors (TLR), hereby mediating cellular tumor immunity [117]. In turn, further investigation studies showed that it mobilizes the endo/lysosomal machinery of melanoma cells, and through melanoma differentiation associated gene-5 (MDA-5) induces self-degradation by (macro) autophagy and apoptosis, following the MDA-5-mediated activation of proapoptotic factor NOXA [118]. Interestingly, at the exact same time, MDA-5 and NOXA were also reported to play a role in interferon-independent apoptosis in human melanoma cells by Besch and collaborators [141]. Not only were these findings meaningful, opening new windows for cancer therapy, but also, in particular in the Damía Tormo studies, was the murine model used very suited, whereupon mice overexpressing hepatocyte growth factor (HGF) and carrying an oncogenic mutation in the cyclin-dependent kinase-4 [(CDK4)^R24C^] developed invasive melanomas in the skin following neonatal exposure to carcinogenics.

While a number of microRNA has been described to play relevant roles in melanoma progression [127], only few *in vitro* studies have reported on the miRNA potential for antimelanoma therapy [119, 120]. However, pertinent therapeutic approaches targeting miRNAs described for other tumor types [142, 143] foretell the potential and the therapeutic window opportunities entailing these nucleic acids in metastatic melanoma.

As an overview of this section, Table 2 presents the therapeutic nucleic acids herein described, and Figure 3 schematically summarizes the different strategies in nucleic acid therapies.

## 7. Conclusions and Future Perspectives

It is of general consensus that the last decade of cancer research significantly expanded our knowledge in tumor development and progression. Unfortunately—similar to the tumor escape shaped by the immune surveillance in an early growth phase—as new therapeutic strategies are applied, tumor cells undergo another round of selection, giving rise to therapy-resistant cells. It is therefore necessary to combine several approaches to attack different paths of tumor escape—a fact that is confirmed by the most significant results reported in studies where such strategies have been used. On this note, nucleic acids deliveries are truly advantageous tools as they allow the systemic delivery of potentially toxic molecules that can be combined with chemotherapy aiming at terminating possible resistant-tumor cells. As an example, recently, Su and collaborators have reported on an antitumor strategy combining TNF-encoding pDNA and chemotherapy [68]. While systemically administered TNF is extremely toxic, in its genetic form, and when reaching specific target cells, TNF revealed to be a powerful antitumor agent. Specific and efficient are indeed key words in this type of targeted approaches, as in suicide gene delivery. It is thus of extreme importance to thoroughly evaluate the target options and to verify the levels of the target molecule in the cells of interest. The activation of possible target-receptors may be desired, such as in the case reported by Poeck et al. [116], but only when not hampering the therapeutic effect by activation of pathways that can lead to cell proliferation/differentiation, enhanced cell migration, or inhibition of apoptosis. As described by Schäfer et al., this can be the case when targeting the epidermal growth factor receptor (EGFR), and it is then desirable to design a ligand that targets the receptor circumventing its activation [144]. On the other hand, the relevance of analyzing the targeted receptor has been well exposed in the short letter of Perris in response to the work published by Davis et al. [138]. To avoid other pitfalls in nanovector development, also the *in vivo* distribution needs to be assessed, preferably by several approaches (e.g., bioluminescence imaging, positron emission tomography (PET), and magnetic resonance imaging (MRI)). To this end, immunohistochemistry studies may be suitable and very convenient to corroborate and support data collected by different means, but also microscopy (mostly *in vitro* but also histochemistry analysis) has had its traps [145].

In summary, already a number of promising nucleic acid strategies exist, and these certainly present less hurdles for delivery than their protein counterpart, as they are smaller, less antigenic, and can bypass certain resistance mechanisms. Nevertheless, further improvements in nonviral targeted delivery appear required to increase the efficacy of such therapies. A small final note regarding the potential of miRNA approaches: microRNA therapies can aim at (1) miRNA upregulation, when the target nucleic acid is enrolled in cell homeostasis and is found silenced in tumor cells; (2) miRNA downregulation by antimiRs, when it is upregulated in tumor cells due to its play in cell proliferation; (3) alternatively, miRNA can also have a role in cell-specific transcription in pDNA vectors containing miRNA binding-sites, allowing the expression of the gene of interest in cells, where the miRNA is silenced. All these assets make miRNA undoubtedly a very elegant and flexible tool.

## Figures and Tables

**Table 1:** Common melanoma-targeting tools: ligands for surface cellular targeting and promoters for tissue-specific transcription.

	Targeting tool	Target	Reference
Ligand	[Nle^4^, dPhe^7^]-*α*-MSH	MC1-R	[74–85]
	cRGD	*α*_v_ *β*_3_	[86–90]
	LDV	*α*_1_ *β*_4_	[91]
	Transferrin	Transferrin receptor	[92]

Promoter	Tyrosinase	—	[93–95]
	MIA	—	[96, 97]

**Table 2:** Different therapeutic strategies against melanoma based on nucleic acids. In the case of DNA-based approaches, a therapeutic gene is delivered to induce a beneficial effect, whereas with RNA based, generally the regimen, is based on silencing of a tumor-active gene. dsRNA mimetic pIC is, as yet, a recent and unique finding, based on polyinosine-polycytidylic acid (pIC) complexed with polyethyleneimine (PEI) that induces tumor cell autophagy and apoptosis. As for the case of micro RNAs (miR), only few *in vitro* studies have been conducted showing the therapeutic potential of the delivery of miRs that were found downregulated in tumor cells.

	Therapeutic/silenced/upregulated gene	Reference
DNA-based approaches	IL-12	[104–106]
	gp100	[107, 108]
	MART-1	[108]
	Tyrosinase	[109]
	IP-10	[110]
	HSV-tk	[111–113]

	N-Cadherin	[114]
	PAR-1	[115]
RNA-based approaches	RRM2	[92]
	Bcl2	[116]
	dsRNA pIC	[117, 118]

miR	Let-7b and miR 199a	[119, 120]
